# Fragile X Mental Retardation Protein Mediates the Effects of Androgen on Hippocampal PSD95 Expression and Dendritic Spines Density/Morphology and Autism-Like Behaviors Through miR-125a

**DOI:** 10.3389/fncel.2022.872347

**Published:** 2022-04-22

**Authors:** Huan Chen, Dan Qiao, Chang Wang, Bohan Zhang, Zhao Wang, Longmei Tang, Yibo Wang, Ran Zhang, Yizhou Zhang, Leigang Song, Hongchun Zuo, Fangzhen Guo, Xia Wang, Sha Li, Huixian Cui

**Affiliations:** ^1^Department of Anatomy, Hebei Medical University, Shijiazhuang, China; ^2^Neuroscience Research Center, Hebei Medical University, Shijiazhuang, China; ^3^Hebei Key Laboratory of Neurodegenerative Disease Mechanism, Shijiazhuang, China; ^4^Department of Epidemiology and Statistics, Hebei Medical University, Shijiazhuang, China; ^5^Clinical Medicine, Hebei Medical University, Shijiazhuang, China; ^6^Department of Child Health (Psychological Behavior), Children’s Hospital of Hebei Province, Shijiazhuang, China

**Keywords:** androgen, FMRP, synaptic plasticity, PSD95, miR-125a

## Abstract

Dysregulated synaptic plasticity is a key feature of neurodevelopmental disorders, including autism. This study investigated whether Fragile X mental retardation protein (FMRP), a selective RNA-binding protein that regulates synaptic protein expression by interacting with miRNAs, mediates the effects of androgens that play an important role in regulating the synaptic plasticity in the hippocampus. Experiments using mouse hippocampal neuron HT22 cells demonstrated that dihydrotestosterone (DHT) increased the expression of postsynaptic density protein 95 (PSD95) by inhibiting FMRP expression. Administration of miR-125a inhibitor upregulated the PSD95 expression and significantly increased the DHT-induced upregulation of PSD95. FMRP knockdown in HT22 cells reduced the expression of miR-125a. Moreover, miR-125a inhibitor upregulated the PSD95 expression in the DHT-treated HT22 cells with FMRP knockdown. Subsequently, the effects of androgen-mediated *via* FMRP in regulating neural behaviors and PSD95 expression and dendritic spines density/morphology were investigated using *Fmr1* knockout (KO) and wild-type littermate (WT) mice. The castration of WT mice reduced the androgen levels, aggravated anxiety and depression, and impaired learning and memory and sociability of mice. DHT supplementation post-castration reversed the alterations in density and maturity of dendritic spines of hippocampal neurons and behavioral disorders in WT mice; however, it did not reveal such effects in *Fmr1* KO mice. Further, immunohistochemical staining and western blotting analyses after knocking down miR-125a revealed similar effects of castration and post-castration DHT supplementation on PSD95 protein expression. These findings clarified that FMRP mediated the effects of DHT through miR-125a in regulating the expression of hippocampal synaptic protein PSD95. This study provides evidence for the neuroprotective mechanism of androgen in PSD95 expression and dendritic spines density/morphology and suggests that treatment interventions with androgen could be helpful for the management of synaptic plasticity disorders.

## Introduction

Autism is a neurodevelopmental disorder characterized by deficits in social communication, language development, and the presence of narrow interest and repetitive behavior (Levy et al., 2009). The incidence of autism has increased markedly (Daniels et al., 2017); however, its etiology is still a mystery. Accumulating evidence suggests that genetic and environmental factors play important roles in its pathogenesis (Chaste and Leboyer, 2012; Bai et al., 2019). For instance, Fragile X Syndrome (FXS) is the most common genetic cause of autism caused by a single gene mutation of the fragile X mental retardation gene 1 (*Fmr1*) encoding the fragile X mental retardation protein (FMRP) (Mila et al., 2018). FMRP is a selective RNA-binding protein highly expressed in many tissues, especially neurons and testicular tissues (Zhang et al., 2014). FMRP can bind 4% mRNA in the mammalian brain (Ashley, Wilkinson et al., 1993) and regulate the translation and expression of target genes related to dendritic development in specific time and space (Banerjee et al., 2018). In many disease models, the loss of function of FMRP leads to an abundance of certain proteins, resulting in dendrite dysplasia and cognitive deficits. The characteristic neuropathological changes of FXS are related to the abnormal development of dendritic spines and synapses (Sokol et al., 2011), which hinders the transformation of filopodia to mature mushroom spines (Jawaid et al., 2018). In addition to *Fmr1*, several other genes at high risk for autism encode synaptic proteins and affect synapse formation, maturation, and synaptic connections (Sugathan et al., 2014; Nia et al., 2020). Therefore, autism is considered as a synaptic dysfunction. Moreover, the impaired synaptic development and function in autism indicate that the neurobiological study of abnormal development of dendritic spines could provide insights into the pathogenesis of autism.

The physiological effects of androgens, the steroid hormones synthesized in the brain, gonads, and adrenal glands in both sexes, play important roles in regulating synaptic plasticity (Tozzi et al., 2019). Our previous study demonstrated that androgens play an important role in maintaining normal hippocampal synaptic plasticity and affect neurobehaviors by changing synaptic density and dendritic spines morphology. Furthermore, the androgen level in autistic children has been reported to be significantly higher than the age-matched healthy children (Majewska et al., 2014; Needham et al., 2021). These studies suggest the neuroplastic effects of abnormal androgen levels on brain development and the incidence and progression of neurological disorders such as autism. However, the underlying mechanism of how these effects are mediated remains elusive.

miRNAs, a class of non-coding single-stranded RNA, play crucial roles in the post-transcriptional regulation of target gene expression (Baby et al., 2020). miRNAs are highly expressed in the nervous system and are involved in a series of physiological functions such as neurodevelopment, dendritic spines morphology, and synaptic plasticity (Hu and Li, 2017). Imbalance in miRNA expression can induce changes in brain circuits and synaptic functions, resulting in some neurological diseases such as autism (Lu et al., 2020) and Alzheimer’s disease (Mai et al., 2019). Several miRNAs targeting the 3′ UTR of PSD95 mRNA have been predicted, including miR-1, miR-103, and miR-125a. miR-125a plays a crucial role in mGluR-mediated PSD95 mRNA translation activation in cultured primary cortical neurons. Further, it has been shown that in neuro2A cells, the transfection of miR-125a, not miR-1 and miR-103, significantly inhibited the expression of f-luciferase-PSD95 UTR (Muddashetty et al., 2011). The associative theory between FMRP and miRNA suggests that the interaction between FMRP and miRNA promotes RNA-induced silencing complex (RISC), which contains Argonaute, and mRNA binding and negatively regulates the translation and expression of synaptic protein mRNA in specific time and space (Muddashetty et al., 2011).

Therefore, in this study, we aimed to test the hypothesis that androgens can regulate the development of dendritic spines and PSD95 abnormalities mediated by FMRP defects through miR-125a. Furthermore, we investigated the pathogenesis of autism from the perspective of the association between FMRP and miRNA.

## Materials and Methods

### Cell Culture

Mouse hippocampal neuron HT22 cells were cultured in Dulbecco’s Modified Eagle Medium (DMEM, cat#: 11965092, Gibco, United States) containing 10% fetal bovine serum (FBS) and 1% penicillin/streptomycin under standard conditions (37°C, 5% CO_2_) in a humidified atmosphere. The cells were digested with 0.25% trypsin at 85–90% confluency. Then, the cells were seeded into 10 cm culture dishes for RNA-binding protein immunoprecipitation. Subsequently, the cells were transferred to 6- or 24-well plates for western blotting, immunofluorescence cytochemical staining, and quantitative reverse transcription-polymerase chain reaction (qRT-PCR) analyses. The cells in the experimental groups were treated with 10 nM dihydrotestosterone (DHT; cat#: A0462, *Tokyo Chemical Industry*, *Japan)* for 36 h, and those in the control (Ctrl) group were treated with equal volume dimethyl sulfoxide (DMSO).

### Animal Castration and Dosing

Two-month-old healthy male *Fmr1* knockout (KO) mice purchased from Jackson Labs (stock # 003025, United States) and wild-type littermate (WT) mice were used. After *Fmr1* KO and WT mice were anesthetized, a small incision was made from the scrotum to remove the testis in the experimental group. The scrotum was cut open and sutured in the Ctrl group without hurting the testis. The experimental groups (DHT group and DHT + anta-125a group) were administered *via* an intraperitoneal (i.p.) injection with DHT (1 mg/kg body weight) after 3 days of castration, and the Ctrl group was injected with equal volume sesame oil for 30 consecutive days. Subsequently, neurobehaviors, PSD95 expression and dendritic spines density/morphology, and the associative regulation of FMRP and miR-125a were evaluated.

The animals were maintained in accordance with the Guide for the Care and Use of Laboratory Animals, and the study was approved by the Animal Experiment Ethics Committee of Hebei Medical University.

### Knockdown of *Fmr1* With RNA Interference

Short hairpin RNA (shRNA) targeting *Fmr1* (5′-CGCACCAAGTTGTCTCTTATA-3′) and negative control (NC) RNA were packaged into lentivirus (Genechem, China). At 30% confluency, the HT22 cells were infected with lentivirus. The culture medium was replaced with fresh medium after 8 h of infection and the cells were cultured for 48 h. Afterward, the stable clones were selected using 4.5 μg/mL puromycin. The knockdown efficiency of FMRP was about 85% by western blotting assay.

### Downregulation of miR-125a in HT22 Cells

Lipofectamine™ 2000 (cat#: 11668-019, Invitrogen, United States) was used to transfect 40 nM miR-125a-5p inhibitor (5′-UCACAGGUUAAAGGGUCUCAGGGA-3′) and NC inhibitor (5′-CAGUACUUUUGUGUAGUACAA-3′) (Genepharma, China) into HT22 cells, respectively. The culture medium was replaced with fresh medium after 4 h of transfection and the cells were cultured for 20 h.

### RNA Immunoprecipitation

The direct binding between FMRP and PSD95 mRNA was verified by RIP. HT22 cells were lysed on ice with a buffer (20 mM Tris–HCl PH 7.5, 150 mM NaCl, 1 mM EDTA, 0.5% NP-40) containing RNA enzyme inhibitors and protease inhibitors. The dynabeads™ Protein G beads (cat#: 10003D, Invitrogen, United States) with 3 μg primary anti-FMRP antibody (cat#: ab17722, Abcom, United States) or rabbit IgG (cat#: *A7016, Beyotime, China*) were incubated for 30 min and mixed with cell lysates for 12 h at 4°C by rotation. Afterward, the sample was washed. The TRIzol reagent containing protein kinase K was added and incubated at 55°C for 30 min to purify and extract RNA for the reverse transcription-polymerase chain reaction (RT-PCR) experiment.

### Reverse Transcription-Polymerase Chain Reaction

The mRNA (2 μg) of HT22 cells extracted by RIP was reverse transcribed into cDNA using the RevertAid First Strand cDNA Synthesis Kit (cat#: K1622, Thermo Scientific, United States). The conditions for reverse transcription were 42°C for 60 min and 70°C for 5 min. Subsequently, the cDNA was used for PCR using the 2 × Taq PCR MasterMix (cat#: KT201, Tiangen, China), and the following primers were used for RT-PCR: postsynaptic density protein 95 (PSD95) forward, 5′-TACCAAAGACCGTGCCAACG-3′ and PSD95 reverse, 5′-CGGCATTGGCTGAGACATCA-3′; GAPDH forward, 5′-CCGGTGCTGAGTATGTCGTG-3′ and GAPDH reverse, 5′-TGGTCATGAGCCCTTCCACA-3′. The cycling conditions were 94°C for 3 min; 38 cycles of 94°C for 30 s, 59°C for 30 s, and 72°C for 30 s; and a final extension at 72°C for 5 min. The RT-PCR products were resolved using agarose gel electrophoresis. Gel images were acquired using the Image Lab system (Bio-Rad, United States), and gray values were analyzed using the Image J software. The relative expression of PSD95 mRNA was calculated with the gray value of GAPDH mRNA as a reference.

### Western Blotting

Radioimmunoprecipitation assay (RIPA) lysis buffer containing phenylmethylsulfonyl fluoride (PMSF) was added to lyse samples, and the proteins were extracted for quantification. Proteins were separated using sodium dodecyl sulfate-polyacrylamide gel electrophoresis (SDS-PAGE) and transferred onto a polyvinylidene fluoride (PVDF) membrane. Then, the membrane was blocked using 5% milk for 2 h and incubated overnight at 4°C with the following primary antibodies: anti-PSD95 (cat#: ab18258, Abcam, United States), anti-FMRP (cat#: ab17722, Abcam, United States), and anti-β-actin (cat#: AC026, ABclonal, China). Subsequently, they were incubated with Dylight™ 800 goat anti-rabbit fluorescent secondary antibody (cat#: 611-145-002, Rockland, United States) at room temperature in the dark for 2 h. Finally, imaging analysis was performed using the Odyssey imaging system (LICOR, United States). The relative expression of the target protein was calculated with the gray value of β-actin as a reference.

### Quantitative Reverse Transcription-Polymerase Chain Reaction

Total miRNA of HT22 cells was extracted and isolated using the miRcute miRNA kit (cat#: DP501, Tiangen, China). cDNA was reverse transcribed using the miRcute enhanced miRNA first-strand cDNA synthesis kit (cat#: KR211, Tiangen, China). The conditions of reverse transcription were 42°C for 60 min and 95°C for 3 min. qRT-PCR was performed using miRcute enhanced miRNA fluorescence quantitation kit (SYBR Green, cat#: FP411, Tiangen, China) on a real-time PCR instrument (Thermo Fisher Scientific, United States). The 2^–ΔΔ^ ^Ct^ method was used for the qRT-PCR analysis. The expression of miR-125a was calculated with the expression of *U6* as an internal reference. miR-125a and U6 primers were obtained from Tiangen Biotech, China.

### Immunofluorescence Cytochemistry

HT22 cells were fixed with 4% paraformaldehyde at room temperature for 15 min, blocked in 10% donkey serum at room temperature for 1 h, and incubated overnight at 4°C with the following primary antibodies: anti-PSD95 (cat#: ab18258, Abcam, United States), anti-FMRP (cat#: ab17722, Abcam, United States). Afterward, the cells were incubated with donkey anti-rabbit fluorescent secondary antibody (cat#: A21207, Invitrogen, United States) at room temperature in the dark for 2 h, counterstained with 4′,6-diamidino-2-phenylindole (DAPI) (cat#: C0065, Solarbio, China) for 10 min, and sealed with *anti*-fluorescence quenching *sealing* tablets (cat#: S2100, Solarbio, China). Images were photographed using a laser confocal microscope (Olympus, Japan), and the average optical density was analyzed using the Image J software (National Institutes of Health, United States).

### Open Field Test

Subsequently, we investigated the involvement of FMRP in androgen regulating neural behaviors using *Fmr1* KO and WT mice. The mice were put into the behavioral laboratory 1 day in advance to adapt to the test environment and reduce animal stress. SMART 3.0 software (Panlab, Spain) was used to divide the central region (25 cm × 25 cm) and the peripheral region of the open field box (50 cm × 50 cm) and record the movement distance and time of the mice in the central and peripheral region within 5 min.

### Morris Water Maze

The classic Morris water maze experiment principle is that rats or mice learn to find hidden platforms with fixed positions and form stable spatial position cognition during multiple training. This spatial cognition is formed by processing spatial information based on external cues (different marks at different positions on the inner wall of cylindrical pool). The circular pool (120 cm in diameter) was divided into four quadrants, and the platform (6 cm in diameter) was placed 1 cm underwater in either quadrant. After 2 days of adaptive training, an orientation *navigation* trial was conducted for five consecutive days. The mice were placed in the water from four quadrants facing the pool wall, and the time of finding the platform was recorded within 60 s, that is, the escape latency. If they did not find the platform within 60 s, the mice were guided to find it and stay for 1 min. On the sixth day, the spatial probe trial was conducted. The platform was removed, and the mice were put into water. The times taken by the mice crossing the position of the platform within 60 s were recorded.

### Forced Swim Test

Transparent glass cylinders (10 cm in diameter) were filled with water up to 20 cm deep. Then, the mice were put into the water-filled cylinder for 6 min, and the total floating time of the mice within 4 min was recorded. Floating time refers to when the mice have no movement other than a slight movement to maintain balance or keep their head above the water.

### Novel Object Recognition Test

Two identical objects were placed on the left and right ends of one sidewall of the open field box, and the mice were placed in a box with their backs to the objects, with the same distance from the two objects, and allowed to explore freely for 10 min. After 24 h, an original object was replaced with a new object of different color and shape. The exploration time of the mice to the two objects was recorded within 5 min, and the exploration distance was 2–3 cm from the objects. The exploration behavior refers to putting the front paw on the object, smelling the object with the nose, licking the object, etc. Holding a pose or climbing on the object without moving is not exploration of the object. The discrimination index, represented by the ratio of the exploration time of the mice to the total exploration time, was calculated.

### Three-Chamber Test

Rectangular open field boxes were separated into three chambers using transparent partitions. The partitions had a door (6 cm × 6 cm) for the free movement of the mice. The mice were habituated to the empty box before the test. To assess the sociability index, one chamber was kept empty and in the third chamber, a new mouse (Stranger) was put. The test mouse was put in the middle chamber and was allowed to explore the chambers freely for 10 min. The time to explore the two cages was recorded at 2–3 cm from the cages. The sociability index was calculated by the ratio of the time taken to explore the “Stranger” to the total time to explore the cages. Next, the social novelty preference index was estimated by putting another new mouse (Novel) in the empty cage, while the “Stranger” was renamed “Familiar.” The time taken by the test mouse to explore the two cages in 10 min was recorded. Then, the social novelty preference index represented by the ratio of the time of exploring the “Novel” mouse to the total time of exploring the cages was calculated.

### Golgi Staining

After the behavioral study, mice were deeply anesthetized and transcardially perfused with 0.9% saline followed by 4% paraformaldehyde. Whole brains were extracted and postfixed in 4% paraformaldehyde for 24 h. The brains were cut from superior colliculus to optic chiasma, stained with Golgi staining kit (cat#: GMS80020.1, Genmed, China) with a fixative solution in the dark for 14 days. Subsequently, they were dehydrated in 30% sucrose solution at 4°Cfor 48 h under dark and sliced into *100 μm* thick sections using oscillating tissue slicers. Brain slices were incubated with staining solution for 30 min at room temperature, followed by incubation in a chromogenic solution for 20 min at room temperature. The stained brain slices were protected from light, dehydrated, and sealed with neutral resin. Secondary and tertiary apical dendritic spines in the hippocampal CAl region were observed and imaged under a 100 × light microscope (Olympus, Japan). On the basis of morphology, spines were classified into the following categories: (i) Thin: spines with a long neck and a visible small head; (ii) Mushroom: big spines with a well-defined neck and a very voluminous head; and (iii) Stubby: very short spines without a distinguishable neck and stubby appearance. After the dendritic spines were visually identified, Fiji software (National Institutes of Health, United States) was used to label them to prevent miscounting. Three brain slices were selected from each mouse and three neurons were selected from each brain slice.

### Immunohistochemical Staining

The mice were perfusion-fixed with PFA, and their brains were removed. The brains were cut from superior colliculus to optic chiasma, conventionally dehydrated, immersed by wax, embedded and sectioned into 5 μm thick slices. After dewaxing and hydration, the sections were subjected to high-pressure antigen repairing. Subsequently, they were blocked and incubated overnight at 4°C with anti-PSD95 antibody (cat#: ab18258, Abcam, United States). Afterward, the sections were incubated with goat anti-rabbit IgG secondary antibody (cat#: SP-9001, ZSGB-BIO, China) for 1 h, horseradish enzyme-labeled streptavidin for 1 h, followed by DAB staining and hematoxylin counterstaining. Images of hippocampal CA1 and CA3 were observed and collected under a 40 × light microscope (Leica, Germany), and the average optical density was analyzed using the Image J software (National Institutes of Health, United States).

### Silencing of miR-125a in Mice

After anesthetized, the mice were put on an electric StereoDrive (NeuroStar, Germany). Their anterior fontanelle and posterior fontanelle were fully exposed, and their lateral ventricles (left ML = −0.95 mm, AP = −0.22 mm, DV = 2.37 mm) were located by mouse brain atlas (Watson, 3rd edition) software. 5 μL of miR-125a antagomir or NC antagomir (20 μM) (miR-125a antagomir sequence: 5′-UCACAGGUUAAAGGGUCUCAGGGA-3′; NC Antagomir sequence: 5′ -CAGUACUUUUGUGUAGUACAA-3′; Genepharma, China) were injected by intracerebroventricular (icv.) injection per day for three consecutive days to knockdown the expression level of miR-125a in the hippocampus of mice. On day 7, the hippocampus or brains were collected for subsequent western blot and immunohistochemical staining.

### Statistical Analysis

All statistical analyses were performed using the SPSS 22.0 statistical software. The experimental results were expressed as mean ± standard deviation (SD). *Data were* subjected to *normality* testing using the Shapiro–Wilk *normality* test. For two-sample comparisons of normally distributed data (*p* > 0.1), Student’s *t*-test was used. Data from multiple groups were subjected to the homogeneity of variances test using *Levene’s test*. One-way analysis of variance (ANOVA) was performed for data with a normal distribution (*p* > 0.1) and homogeneity of variance (*p* > 0.1), and *post hoc* multiple comparisons were performed with the *SNK-q* test. Differences were considered statistically significant at *p* < *0.05.*

## Results

### Effects of DHT on PSD95 Protein and FMRP in HT22 Cells

**FIGURE 1:**
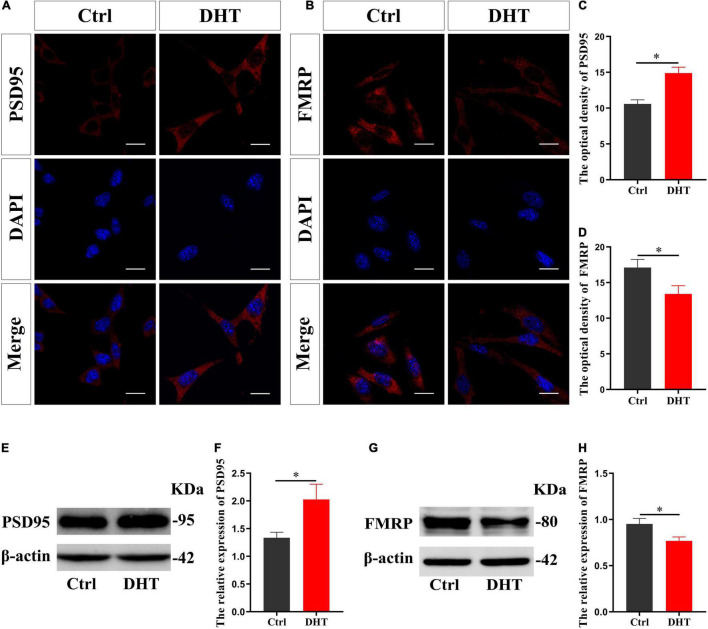
Effects of DHT on PSD95 protein and FMRP in HT22 cells. **(A,C)** Immunofluorescence cytochemistry for PSD95 expression induced by DHT in HT22 cells. **(B,D)** Immunofluorescence cytochemistry for FMRP expression induced by DHT in HT22 cells. **(E,F)** Western blotting for PSD95 expression induced by DHT in HT22 cells. **(G,H)** Western blotting for FMRP expression induced by DHT in HT22 cells. Scale bars = 20 μm (*p < 0.05, *n* = 5).

Immunofluorescence cytochemical staining results showed that the fluorescence intensity of PSD95 in the DHT group was higher than that in the Ctrl group [*t*_(8)_ = −9.739, *p* < 0.05, Cohen’s *d* = 6.887] (Figures 1A,C). On the contrary, the fluorescence intensity of FMRP in the DHT group was lower than that in the Ctrl group [*t*_(8)_ = 5.080, *p* < 0.05, Cohen’s *d* = 3.592] (Figures 1B,D). These results were further validated using western blotting, which showed that the expression of PSD95 in the DHT group was higher than that in the Ctrl group [*t*_(8)_ = -5.360, *p* < 0.05, Cohen’s *d* = 3.790] (Figures 1E,F). Conversely, the expression of FMRP in the DHT group was lower than that in the Ctrl group [*t*_(8)_ = 5.842, *p* < 0.05, Cohen’s *d* = 4.131] (Figures 1G,H). DHT upregulated the expression of synaptic protein PSD95 and downregulated the expression of FMRP, affecting both proteins, which laid a foundation for us to investigate whether FMRP mediates DHT upregulated PSD95.

### FMRP Regulates DHT Affecting the Expression of PSD95 Protein in HT22 Cells

**FIGURE 2:**
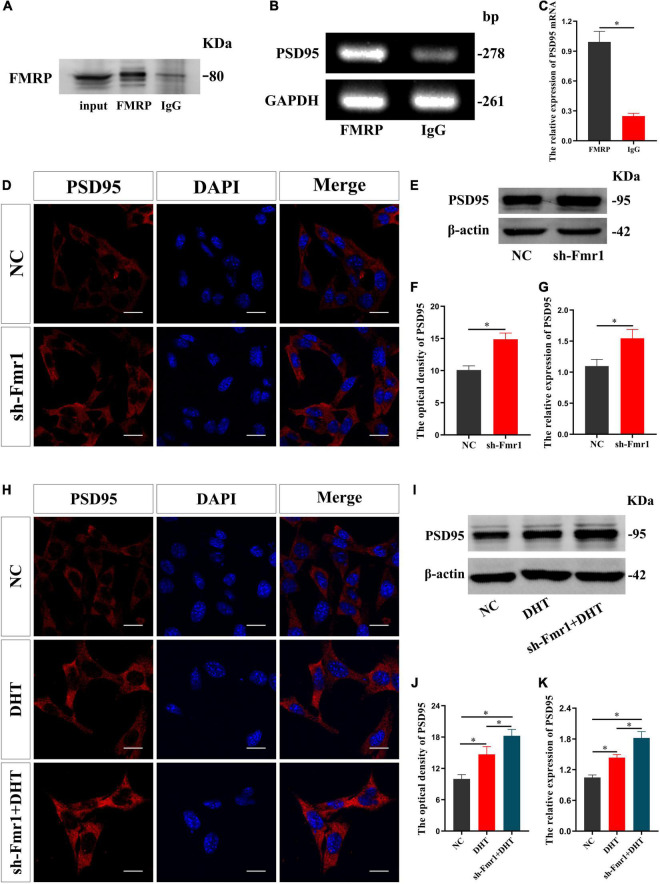
FMRP regulates DHT-induced expression of PSD95 protein in HT22 cells. **(A–C)** RNA immunoprecipitation for the interaction between FMRP and PSD95 mRNA in HT22 cells. **(D**,**F)** Immunofluorescence cytochemistry for PSD95 expression in HT22 cells pre-treated with NC or sh-Fmr1. **(E**,**G)** Western blotting for PSD95 expression in HT22 cells pre-treated with NC or sh-Fmr1. **(H**,**J)** Immunofluorescence cytochemistry for PSD95 expression induced by DHT *via* FMRP in HT22 cells. **(I**,**K)** Western blotting for PSD95 expression induced by DHT *via* FMRP in HT22 cells. Scale bars = 20 μm (*p < 0.05, *n* = 5).

RIP revealed that the input and FMRP groups demonstrated specific binding to the FMRP antibody, wherein only non-specific binding to the control IgG group was observed (Figure 2A). The enrichment of PSD95 mRNA in the FMRP group was higher than that in control IgG group [*t*_(8)_ = 15.447, *p* < 0.05, Cohen’s *d* = 10.923] (Figures 2B,C). We also infected HT22 cells with sh-Fmr1 lentivirus to knockdown FMRP and detected its effect on PSD95. Immunofluorescence cytochemical staining showed that the fluorescence intensity of PSD95 in the sh-Fmr1 group was higher than that in the NC group [*t*_(8)_ = −9.209, *p* < 0.05, Cohen’s *d* = 6.512] (Figures 2D,F). Western blotting results showed that the expression of PSD95 in the sh-Fmr1 group was higher than that in the NC group [*t*_(8)_ = -5.543, *p* < 0.05, Cohen’s *d* = 3.919] (Figures 2E,G). In addition, immunofluorescence cytochemical staining showed that fluorescence intensity of PSD95 was significantly different in all groups [*F*_(2,12)_ = 1.517, *p* < 0.05, η^2^ = 0.908]. The fluorescence intensity of PSD95 in the DHT group was higher than that in the NC group but lower than that in the sh-Fmr1 + DHT group (Figures 2H,J). Consistent with these results, western blotting also demonstrated higher expression of PSD95 in the sh-Fmr1 + DHT group than that in the DHT and NC groups [*F*_(2,12)_ = 2.159, *p* < 0.05, η^2^ = 0.947] (Figures 2I,K).

### FMRP and miR-125a Associatively Regulate the Effects of DHT on PSD95 Protein in HT22 Cells

**FIGURE 3:**
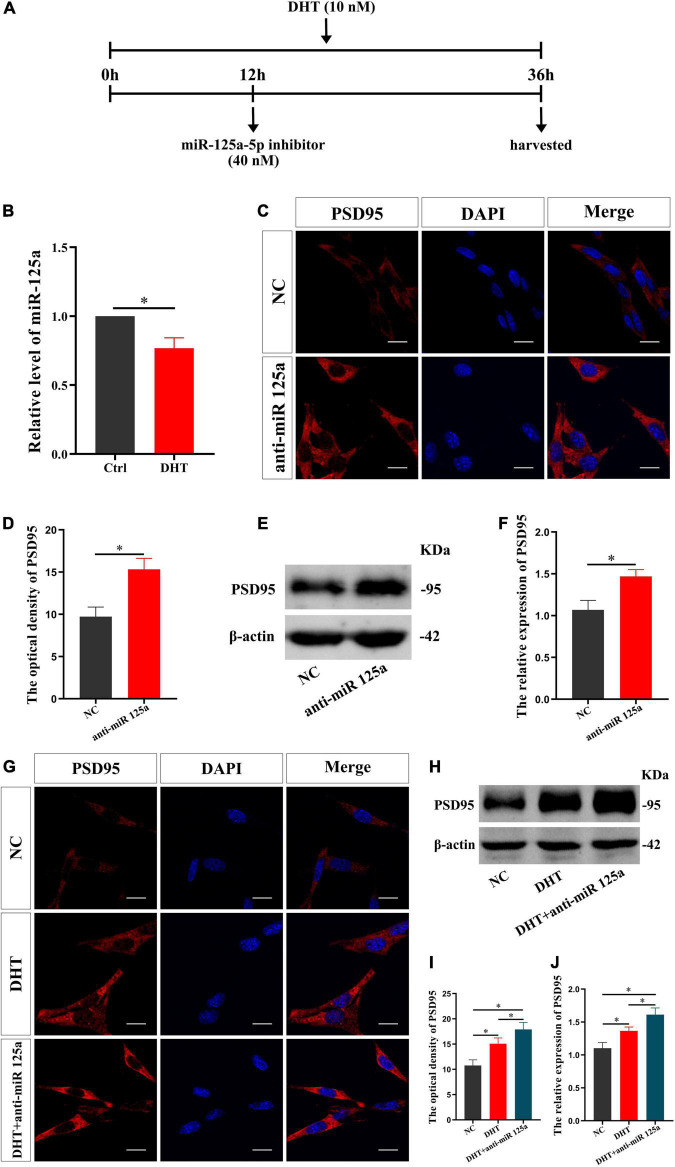
miR-125a regulates DHT affecting the expression of PSD95 protein in HT22 cells. **(A)** Experimental procedure. HT22 cells were treated with DHT (10 nM) and miR-125a-5p inhibitor (40 nM). **(B)** qRT-PCR for miR-125a induced by DHT in HT22 cells. **(C**,**D)** Immunofluorescence cytochemistry for PSD95 expression in HT22 cells pre-treated with NC inhibitor or miR-125a-5p inhibitor. **(E,F)** Western blotting for PSD95 expression in HT22 cells pre-treated with NC or miR-125a-5p inhibitors. **(G**,**I)** Immunofluorescence cytochemistry for PSD95 expression induced by DHT *via* miR-125a in HT22 cells. **(H,J)** Western blotting for PSD95 expression induced by DHT *via* miR-125a in HT22 cells. Scale bars = 20 μm (*p < 0.05, *n* = 5).

FMRP knockdown enhances DHT to upregulate the expression of synaptic protein PSD95, which verifies our hypothesis. Then, to determine whether miR-125a is a downstream molecule of FMRP and mediates the effects of DHT on PSD95 in HT22 cells, we conducted a series of analyses (administration process, Figure 3A). First, the effect of DHT on the expression of miR-125a was assessed using qRT-PCR. The expression of miR-125a in the DHT group was lower than that in the Ctrl group [*t*_(4)_ = −7.133, *p* < 0.05, Cohen’s *d* = 5.044] (Figure 3B). Second, immunofluorescence cytochemical staining to estimate the effect of miR-125a on PSD95 revealed that the fluorescence intensity of PSD95 in the anti-miR 125a group was higher than that in the NC group [*t*_(8)_ = −7.216, *p* < 0.05, Cohen’s *d* = 5.102] (Figures 3C,D). In addition, western blotting demonstrated higher expression of PSD95 in the anti-miR 125a group than in the NC group [*t*_(8)_ = −6.455, *p* < 0.05, Cohen’s *d* = 4.564] (Figures 3E,F).

Next, we investigated whether miR-125a was involved in regulating the DHT-induced expression of PSD95. The fluorescence intensity of PSD95 was significantly different among all groups [*F*_(2,12)_ = 0.090, *p* < 0.05, η^2^ = 0.879]. The fluorescence intensity of PSD95 in the DHT group was higher than that in the NC group. Meanwhile, the fluorescence intensity of PSD95 in the DHT + anti-miR 125a group was higher than that in the DHT group (Figures 3G,I). Western blotting [*F*_(2,12)_ = 0.378, *p* < 0.05, η^2^ = 0.887] also revealed similar results of higher expression of PSD95 in the DHT + anti-miR 125a group than in the DHT and NC groups (Figures 3H,J).

**FIGURE 4:**
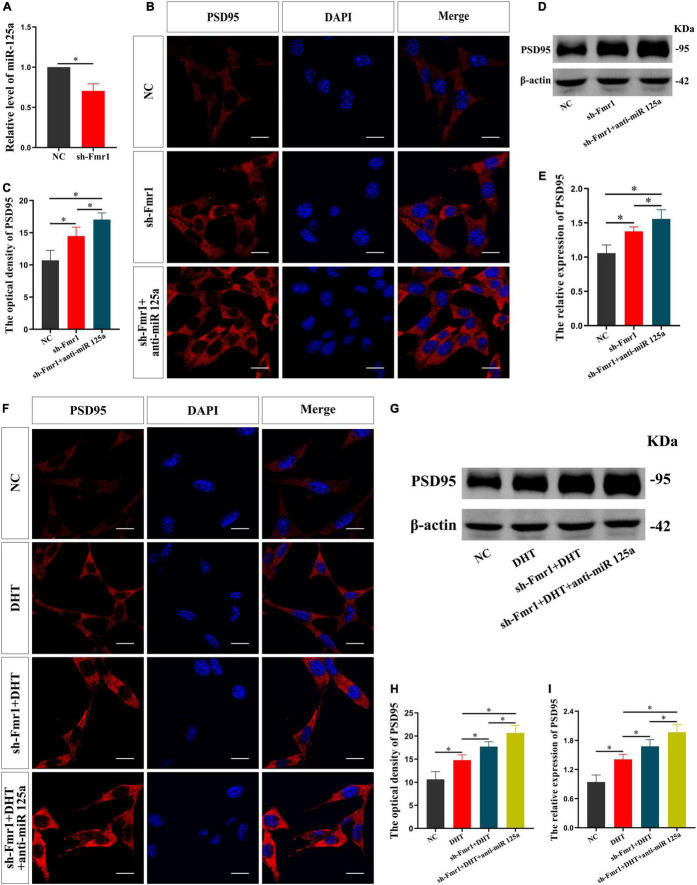
FMRP mediates the effects of DHT on PSD95 through miR-125a in HT22 cells. **(A)** qRT-PCR for miR-125a induced by FMRP in HT22 cells. **(B,C)** Immunofluorescence cytochemistry for PSD95 expression in HT22 cells pre-treated with NC or sh-Fmr1 and with NC inhibitor or miR-125a-5p inhibitor. **(D,E)** Western blotting for PSD95 expression in HT22 cells pre-treated with NC or sh-Fmr1 and NC inhibitor or miR-125a-5p inhibitor. **(F**,**H)** Immunofluorescence cytochemistry for PSD95 expression induced by DHT in HT22 cells pre-treated with NC or sh-Fmr1 and with NC inhibitor or miR-125a-5p inhibitor. **(G**,**I)** Western blotting for PSD95 expression induced by DHT in HT22 cells pre-treated with NC or sh-Fmr1 and NC inhibitor or miR-125a-5p inhibitor. Scale bars = 20 μm (*p < 0.05, *n* = 5).

Subsequently, the effect of FMRP knockdown on the expression of miR-125a was investigated. qRT-PCR showed a lower expression of miR-125a in the sh-Fmr1 group than in the NC group [*t*_(4)_ = −7.481, *p* < 0.05, Cohen’s *d* = 7.481] (Figure 4A). Further, we investigated the involvement of miR-125a in regulating FMRP-mediated PSD95 expression. Immunofluorescence cytochemical staining demonstrated significantly different fluorescence intensity of PSD95 in all groups [*F*_(2,12)_ = 0.476, *p* < 0.05, η^2^ = 0.824]. Particularly, the fluorescence intensity of PSD95 in the sh-Fmr1 + anti-miR 125a group was higher than that in the sh-Fmr1 and NC groups (Figures 4B,C). Furthermore, the results of western blotting [*F*_(2,12)_ = 2.708, *p* < 0.05, η^2^ = 0.816] were consistent with the results of immunofluorescence cytochemical staining (Figures 4D,E).

Finally, we investigated whether FMRP and miR-125a associatively regulate the effects of DHT on PSD95 expression. Immunofluorescence cytochemical staining results showed that the fluorescence intensity of PSD95 was statistically different in all groups [*F*_(3,16)_ = 0.329, *p* < 0.05, η^2^ = 0.899]. The fluorescence intensity of PSD95 in the DHT group was higher than that in the NC group, whereas that in the sh-Fmr1 + DHT + anti-miR-125a group was higher than the sh-Fmr1 + DHT group (Figures 4F,H). Western blotting revealed similar results [*F*_(3,16)_ = 0.608, *p* < 0.05, η^2^ = 0.902] as those of immunofluorescence cytochemical staining (Figures 4G,I).

### Effects of Castration and DHT Supplementation on the Behaviors of *Fmr1* KO Mice

**FIGURE 5:**
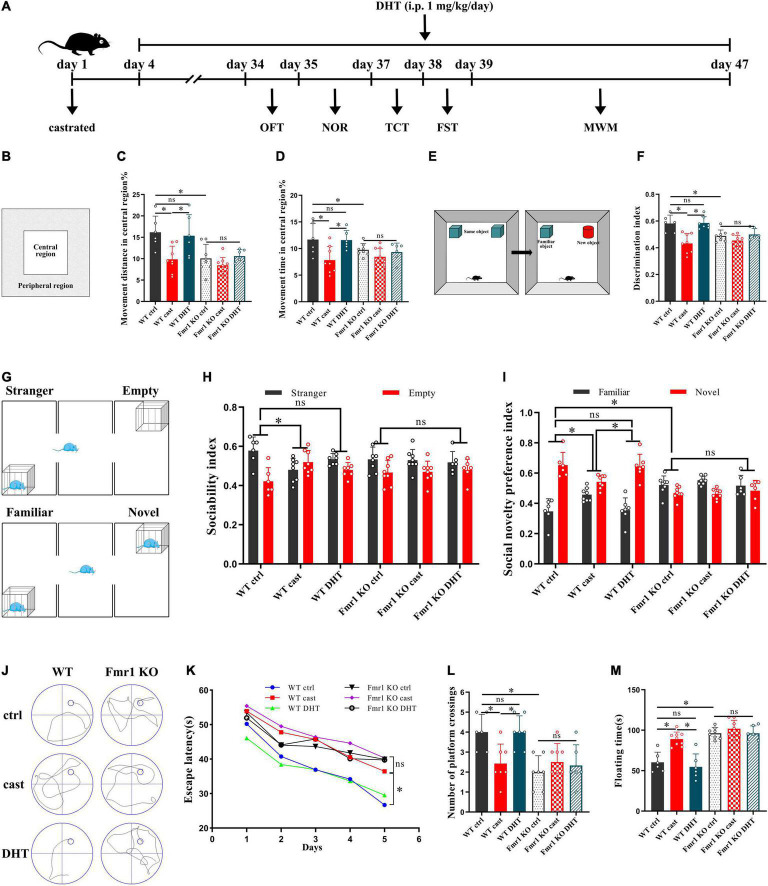
Effects of castration and DHT supplementation on the behaviors of *Fmr1* KO mice. **(A)** Experimental procedure. Mice were treated with castration, DHT (i.p., 1 mg/kg/day), and behavioral tests. **(B)** Schematic diagram of the OFT. **(C,D)** OFT was performed to assess exploratory behavior. **(E)** Schematic diagram of the NOR. **(F)** NOR was performed to assess memory retention. **(G)** Schematic diagram of the TCT. **(H,I)** TCT was performed to test for sociability and social novelty preference. **(J)** Trajectories of the MWM (the 5th day). **(K,L)** MWM was performed to test for learning and memory. **(M)** FST was performed to assess depressive-like behavior (*p < 0.05).

We then tested the effect of DHT on the behaviors in WT and *Fmr1* KO mice (Figure 5A). The OFT showed that there were statistical differences in the percentage of movement distance [*F*_(5,37)_ = 1.702, *p* < 0.05, η^2^ = 0.480] and the percentage of movement time [*F*_(5,37)_ = 1.024, *p* < 0.05, η^2^ = 0.447] in central region in all groups. The percentage of movement distance and time of the *Fmr1* KO Ctrl and WT castration group in the central region was lower than that in the WT Ctrl group. DHT supplementation increased the movement distance and time of WT mice in the central region. However, neither castration nor DHT supplementation significantly affected the movement distance and time of the *Fmr1* KO mice in the central region (Figures 5B–D).

The NOR test revealed significant differences in discrimination index in all groups [*F*_(5,38)_ = 1.485, *p* < 0.05, η^2^ = 0.586]. The discrimination index of mice in the *Fmr1* KO Ctrl and WT castration groups was lower than that in the WT Ctrl group. DHT supplementation increased the discrimination index of WT mice. However, neither castration nor DHT supplementation affected the discrimination index of *Fmr1* KO mice (Figures 5E,F).

The TCT also showed significant differences in sociability [*F*_(5,37)_ = 0.777, *p* < 0.05, η^2^ = 0.229] and social novelty preference indices [*F*_(5,37)_ = 1.033, *p* < 0.05, η^2^ = 0.674] in all groups. The sociability index of the WT castration group and the social novelty preference indices of the *Fmr1* KO Ctrl and WT castration groups were lower than that of the WT Ctrl group. DHT supplementation restored sociability and social novelty preference in WT mice. However, neither castration nor DHT supplementation affected sociability and social novelty preference in *Fmr1* KO mice (Figures 5G–I).

The MWM test demonstrated significant differences in escape latency on day 5 [*F*_(5,36)_ = 0.882, *p* < 0.05, η^2^ = 0.815] and number of platform crossing [*F*_(5,36)_ = 0.206, *p* < 0.05, η^2^ = 0.484] in all groups. Compared with the mice in the WT Ctrl group, the escape latency of mice in the *Fmr1* KO Ctrl and WT castration groups was increased, whereas the number of platform crossings was decreased. DHT supplementation restored the learning and memory ability of the WT mice but not those in the *Fmr1* KO mice (Figures 5J–L).

The floating time in all groups differed significantly [*F*_(5,37)_ = 1.032, *p* < 0.05, η^2^ = 0.761]. The FST test demonstrated that the floating time of mice in the *Fmr1* Ctrl and WT castration groups was higher than that in the WT Ctrl group. Although DHT supplementation reduced floating time in WT mice, neither castration nor DHT supplementation affected the floating time in *Fmr1* KO mice (Figure 5M).

### Effects of Castration and DHT Supplementation on Dendritic Spines of *Fmr1* KO Mice

**FIGURE 6:**
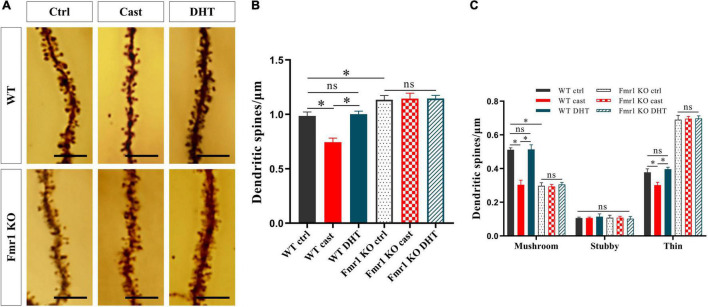
Effects of castration and DHT supplementation on dendritic spines of *Fmr1* KO mice. **(A)** Golgi staining of hippocampal CA1 pyramidal neurons for dendritic spine counting after behaviors. Scale bars = 5 μm. **(B)** Quantification of dendritic spine density in hippocampal CA1 pyramidal neurons calculated as the number of spines per 1 μm of dendrite. **(C)** Quantification of mushroom, stubby, and thin dendritic spine density in hippocampal CA1 pyramidal neurons calculated as the number of spines per 1 μm of the dendrite (*p < 0.05, *n* = 5).

We studied the effect of DHT on dendritic spines of hippocampal neurons in *Fmr1* KO and WT mice using Golgi staining after the behavioral experiments. The density of dendritic spines of the hippocampus was significantly different among all groups [*F*_(5,24)_ = 0.550, *p* < 0.05, η^2^ = 0.948]. The Golgi staining revealed a higher density of dendritic spines in the *Fmr1* KO Ctrl and WT castration groups than in the WT Ctrl group (Figures 6A,B). DHT supplementation increased the density of dendritic spines in WT mice. However, neither castration nor DHT supplementation affected the density of dendritic spines in *Fmr1* KO mice (Figures 6A,B).

The density of mushroom dendritic spines [*F*_(5,24)_ = 1.413, *p* < 0.05, η^2^ = 0.973] and the density of thin dendritic spines [*F*_(5,24)_ = 0.638, *p* < 0.05, η^2^ = 0.992] differed significantly in all groups; whereas no significant difference in the density of stubby dendritic spines was observed [*F*_(5,24)_ = 2.254, *p* < 0.05, η^2^ = 0.079]. Compared with the WT Ctrl group, the density of mushroom dendritic spines in the *Fmr1* KO Ctrl group decreased, and that of the thin dendritic spines increased. The density of mushroom and thin dendritic spines in the WT castration group was lower than that in the WT Ctrl group. DHT supplementation increased the density of mushroom and thin dendritic spines in WT mice; however, no changes were observed in *Fmr1* KO mice either after castration or after DHT supplementation (Figures 6A,C).

### FMRP and miR-125a Associatively Regulate the Effects of DHT on PSD95 Protein in *Fmr1* KO Mice

**FIGURE 7:**
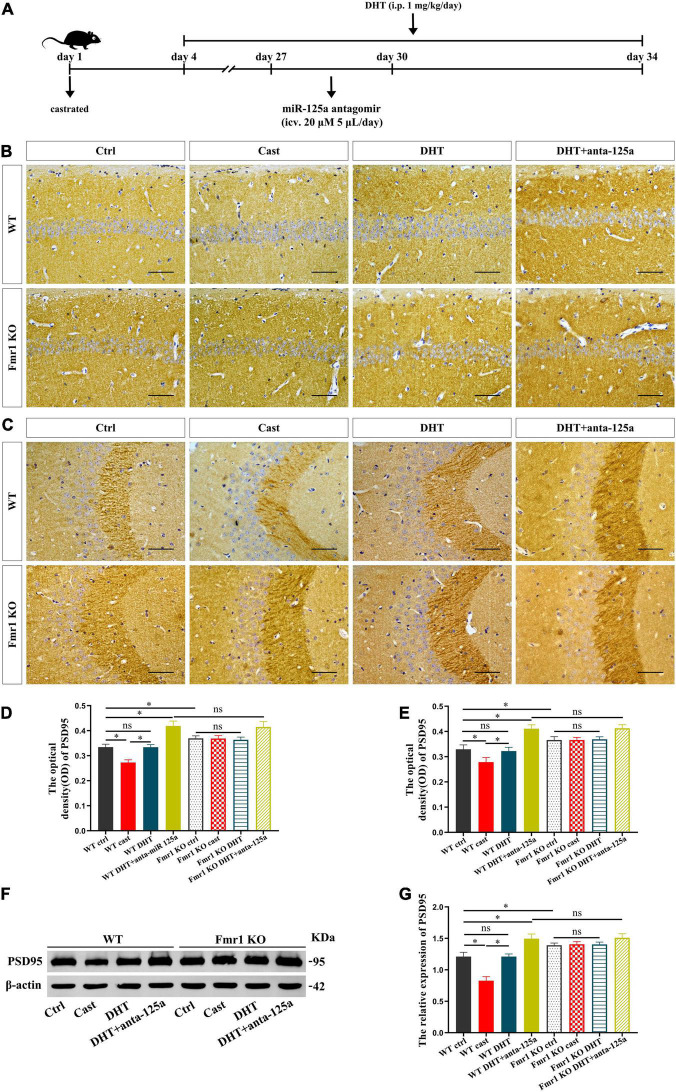
FMRP and miR-125a associatively regulate the effects of DHT on PSD95 protein in *Fmr1* KO mice. **(A)** Experimental procedure. Mice were treated with castration and DHT (i.p. 1 mg/kg/day), and miR-125a antagomir (icv. 20 μm, 5 μL/day). **(B,C)** Immunohistochemical staining for PSD95 of hippocampal CA1 and CA3 pyramidal neurons induced by DHT pre-treated with miR-125a antagomir or NC antagomir. **(D,E)** Graphs show the average optical density values of PSD95 protein of hippocampal CA1 and CA3 pyramidal neurons. **(F)** Western blotting for PSD95 of hippocampus induced by DHT pre-treated with miR-125a antagomir or NC antagomir. **(G)** Graphs show the relative expression of PSD95 protein of the hippocampus. Scale bars = 50 μm (*p < 0.05, *n* = 5).

We then verified whether FMRP mediates the effects of DHT on synaptic protein PSD95 in *Fmr1* KO and WT mice through miR-125a, and whether the results were consistent with cells (Figure 7A). Immunohistochemical staining demonstrated significant differences in the expression of PSD95 in hippocampal CA1 region [*F*_(7,32)_ = 1.494, *p* < 0.05, η^2^ = 0.926] and CA3 region [*F*_(7,32)_ = 0.414, *p* < 0.05, η^2^ = 0.914] in all groups. PSD95 expression in hippocampal CA1 and CA3 regions of the WT castration group was decreased compared with that in the hippocampal CA1 and CA3 regions of the WT Ctrl group. However, DHT supplementation recovered the PSD95 expression of WT mice, its expression in *Fmr1* KO mice did not change after castration or DHT supplementation. Notably, antagomir treatment considerably increased the expression of PSD95 in hippocampal CA1 and CA3 regions of WT DHT + anta-125a and *Fmr1* KO DHT + anta-125a groups than in the hippocampal CA1 and CA3 regions of the WT Ctrl and WT DHT groups (Figures 7B–E).

These results were further confirmed by western blotting. The expression of PSD95 in hippocampus was significantly different in all groups [*F*_(7,32)_ = 1.172, *p* < 0.05, η^2^ = 0.947]. The expression of PSD95 in the hippocampus of the WT castration group was lower than that in the hippocampus of the WT Ctrl group. DHT supplementation recovered the PSD95 expression in WT mice; however, there was no effect of castration or DHT supplementation on the expression of PSD95 in *Fmr1* KO mice. Moreover, antagomir treatment increased the expression of PSD95 in the hippocampus of WT DHT + anta-125a and *Fmr1* KO DHT + anta-125a groups compared with that in the hippocampus of WT Ctrl and WT DHT groups (Figures 7F,G).

**FIGURE 8:**
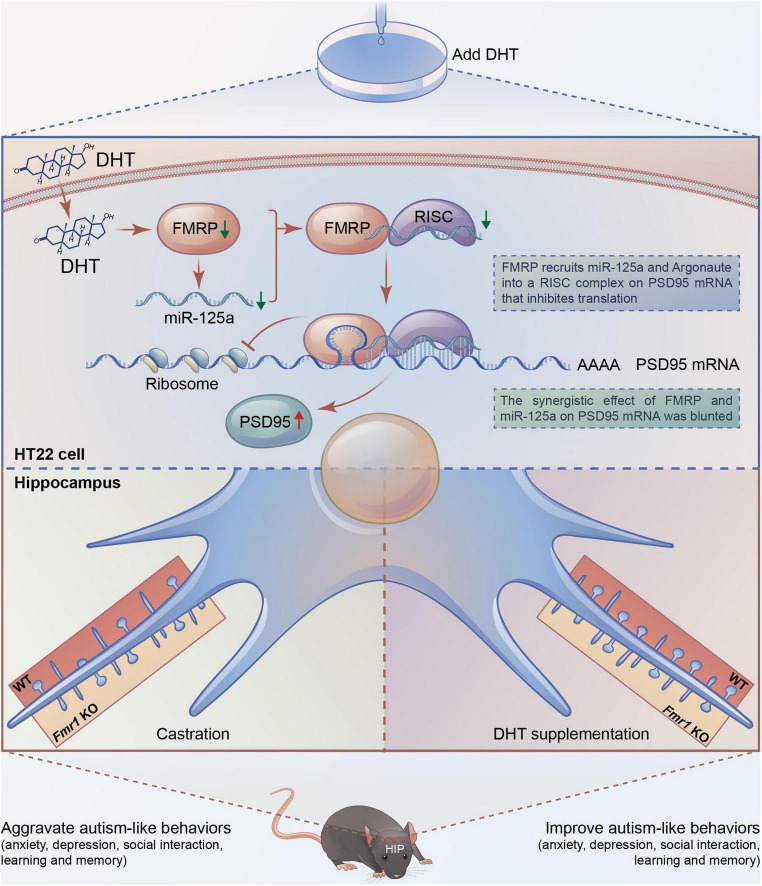
Integration of FMRP and miR-125a associatively regulating the effects of DHT on synaptic plasticity and autism-like behaviors.

According to the result, we draw the integration diagram of FMRP and miR-125a associatively regulating the effects of DHT on PSD95 expression and dendritic spines density/morphology and autism-like behaviors (Figure 8).

## Discussion

Autism is a complex neurodevelopmental disorder commonly seen in children (Silverman et al., 2010). Since its first recognition and naming in 1943 (Kanner, 1943), the understanding of the etiology of autism has been deepened with extensive research conducted, starting from the initial biology to psychology and then to the present genetics. Accumulating evidence shows that FXS is the most common genetic intellectual disorder and the most major single-gene genetic disorder causing autism (Mila et al., 2018). It is caused by the abnormal methylation of CpG island upstream caused by unstable amplification of CGG repeats in the 5′ untranslated region (UTR) of *Fmr1* gene on the X chromosome, resulting in *Fmr1* transcriptional silencing and loss of protein product FMRP (Sutcliffe et al., 1992). FMRP is highly expressed in the mammalian central nervous system and is located in the cell body and dendrites of neurons (Zhang et al., 2015). As an RNA-binding protein, FMRP directly binds to the mRNAs of a large number of genes related to neural development and remodeling in a variety of ways and regulates protein synthesis and function, thus affecting the development of neuronal dendrites and dendritic spines, and synaptic plasticity (Jin and Warren, 2003; Vita et al., 2021). Zhang et al. (Reeve et al., 2008; Yao et al., 2011) found that dFMRP encoded by Drosophila *dfmr1* gene (homologous gene of human *Fmr1* gene) can regulate synaptic development. The studies on cadaveric brain tissues of FXS patients found abnormal development of dendritic spines, and an increase of dendritic spines on the apical and basal dendrites of neurons was observed in multiple cortical regions (Hinton et al., 1991). Loss of FMRP may result in dysplasia or impaired elimination of dendritic spines, providing important information to unravel the molecular mechanism of abnormal development of dendritic spines and synaptic dysfunction.

Sex hormones play an important role in brain structural (Hara et al., 2015; Hyer et al., 2018) and functional development (Okamoto et al., 2012) and sexual dimorphism (Marrocco and McEwen, 2016). Androgen levels were higher in autistic people than non-autistic (Majewska et al., 2014; Needham et al., 2021). But there is a great difference in behavior between males and females with autism. Males with autism show more explicit behavioral problems, such as aggressive behavior (Neuhaus et al., 2022), excessive activity, stereotypical behavior, and narrow interests (Knutsen et al., 2019). Women with autism had more severe internalized symptoms, such as anxiety, depression (Werling and Geschwind, 2013), social problems, attention problems, and thought problems (Holtmann et al., 2007), but there were no gender differences in core autism symptoms in childhood, suggesting that abnormal sex hormone levels may be responsible for differences in behavior and brain structure.

Androgens play a role in regulating synaptic plasticity (Tozzi et al., 2019). The density of dendritic spines in the CA1 region of the hippocampus decreased significantly after castration in mice. Androgen supplementation increased the density of dendritic spines to the same level as that of animals with intact gonads (Li et al., 2015). Further, the effect of androgens on hippocampal synaptic plasticity does not depend on estrogen conversion because aromatase inhibitors do not affect androgens. In contrast, androgen antagonists attenuated the effect of androgen on dendritic spines. These findings suggest that androgens play an important role in maintaining the normal density of dendritic spines in the male hippocampus. However, the effects of androgens on synaptic plasticity are different in different animal models and different types of synapses (Moser et al., 1994; Skucas et al., 2013; Li et al., 2015). To explore the regulatory role and importance of androgens in the development of dendritic spines and abnormal behaviors in the brain of autistic individuals, in this study, a non-aromatizable androgen DHT was used as an intervening drug.

PSD95 is one of the most important proteins in the postsynaptic density (PSD) family. Its main function is to maintain the balance between excitatory and inhibitory synapses (Smith et al., 2017) and to participate in synapse development (Lambert et al., 2017) and synaptic plasticity (Zeng et al., 2016; Wang et al., 2021) through signal transduction and integration (Tulodziecka et al., 2016; Ren et al., 2019). Neurobiological studies have shown that autism is not a localized injury to a specific brain region but a structural abnormality of the entire brain during early development (Courchesne et al., 2005). The most common neuroanatomical feature of autism is the excessive growth and accelerated brain volume development in early childhood, leading to neural network connections changes (Courchesne et al., 2003). Studies have also demonstrated an imbalance of excitatory/inhibitory synapses in the central nervous system of autism patients (Jeckel et al., 2021). For example, Tsai et al. (2012) have shown that multiple autism-related genes can promote the elimination of excitatory synapses by regulating proteasome degradation of PSD95, which is conducive to improving synaptic plasticity in autism. Further, it has also been shown that the downregulation of postsynaptic FMRP leads to increased inhibitory synapses and decreased excitatory synapses. These results in abnormal synaptogenesis, the imbalance of the ratio of excitatory and inhibitory neurons, and alteration in the development of neural network connections (Yang et al., 2021).

In this study, we found that DHT increased PSD95 expression and decreased FMRP expression. After *Fmr1* knockdown in HT22 cells, the expression of PSD95 protein was elevated. However, when the HT22 cells with *Fmr1* knockdown were treated with DHT intervention, the expression of PSD95 protein was higher than that of the DHT alone intervention group, suggesting that FMRP was involved in regulating DHT affecting the expression of PSD95 in HT22 cells. Experimental results of mice demonstrated that castration decreased the androgen level, expression of PSD95, the density of dendritic spines, altered the maturity of dendritic spines, aggravated anxiety and depression, impaired learning memory function, and social ability in WT mice. However, these effects were recovered with DHT supplementation in WT mice. On the contrary, in *Fmr1* KO mice, neither castration nor DHT supplementation affected PSD95 protein expression, density, maturity of dendritic spines, and neurobehaviors.

In this study, we demonstrated that DHT decreased the expression of miR-125a in HT22 cells, and the expression of PSD95 protein increased after inhibition of miR-125a. In addition, DHT significantly upregulated the expression of PSD95 protein in the hippocampus of WT mice silenced miR-125a by antagomir injected into the lateral ventricle compared with the groups without miR-125a intervention. These results demonstrated that both FMRP and miR-125a are involved in DHT-induced PSD95 protein expression. These findings indicated that a mechanism of interaction between FMRP and miR-125a might be important for understanding the neurodevelopmental disorders such as autism caused by the imbalance of the post-transcriptional translation process due to abnormal androgen levels.

The study provided further insights into such interactions. We showed that *Fmr1* knockdown reduced the expression of miR-125a in HT22 cells, and the expression of PSD95 protein in the *Fmr1* and miR-125a knockdown group was significantly higher than that in the *Fmr1* knockdown group. These results suggest that miR-125a is involved in the process of FMRP regulating PSD95 protein translation. However, these results could not precisely clarify the interaction mechanism between FMRP and miR-125a. It has been reported that FMRP affects the rapid and reversible regulation of the miR-125a-mediated translation process, and FMRP phosphorylation promotes the formation of miR-125a-AGO2 translation repressor complex on PSD95 mRNA. Conversely, FMRP dephosphorylation activates the translation process (Muddashetty et al., 2011). Based on the associative theory between FMRP and miRNA, we studied the effect of DHT on PSD95 protein expression. It is noteworthy that our study showed that DHT significantly upregulated the expression of PSD95 in *Fmr1* and miR-125a knockdown group in HT22 cells compared with the *Fmr1* knockdown group. These observations could be explained by the fact that although *Fmr1* knockdown significantly downregulated the expression of FMRP in HT22 cells (knockdown efficiency was about 85%), it was partially expressed. Conversely, in *Fmr1* KO mice, FMRP was completely downregulated; therefore, DHT-induced upregulation in PSD95 protein expression, density and maturity of dendritic spines, and neurobehaviors mediated *via* FMRP was inhibited. These results indicate that FMRP mediated the effects of DHT on the expression of PSD95 protein and dendritic spines density/morphology through miR-125a.

## Conclusion

In conclusion, a series of analyses were conducted to understand the effects of androgens regulating the expression of PSD95 and the maturity of dendritic spines and autism-like behaviors. The analyses revealed that FMRP targeting PSD95 mRNA inhibits the translation of PSD95 protein through miR-125a. This study provides a basis for further studies to understand the interaction between miRNA and FMRP and its role in the dynamic and bidirectional control of excitatory postsynaptic protein translation and synthesis that affects the structure and function of synapses, which has broad significance for neurodevelopmental disorders such as autism, including FXS with abnormal androgen levels. Collectively, we suggest that androgen intervention strategies can be considered to prevent and treat neurodevelopmental disorders; however, further studies should ascertain the effects.

## Data Availability Statement

The original contributions presented in the study are included in the article/supplementary material, further inquiries can be directed to the corresponding author/s.

## Ethics Statement

The animal study was reviewed and approved by Hebei Medical University Laboratory Animal Welfare and Ethics Committee.

## Author Contributions

HC and DQ performed the experimental phase, methodology, data curation, and writing-original draft. CW, BZ, ZW, YW, and RZ performed the experimental phase. YZ, LS, HZ, FG, and XW contributed to the investigation and visualization. LT contributed to the statistical support. SL and HC designed the study, writing-review, editing, project administration, and funding acquisition. All authors contributed to the article and approved the submitted.

## Conflict of Interest

The authors declare that the research was conducted in the absence of any commercial or financial relationships that could be construed as a potential conflict of interest.

## Publisher’s Note

All claims expressed in this article are solely those of the authors and do not necessarily represent those of their affiliated organizations, or those of the publisher, the editors and the reviewers. Any product that may be evaluated in this article, or claim that may be made by its manufacturer, is not guaranteed or endorsed by the publisher.
